# Factors influencing adoption of e-learning in healthcare: integration of UTAUT and TTF model

**DOI:** 10.1186/s12911-022-02060-9

**Published:** 2022-12-09

**Authors:** Mohammadhiwa Abdekhoda, Afsaneh Dehnad, Javad Zarei

**Affiliations:** 1 Department of Medical Library and Information Sciences, School of Health Management and Medical Informatics, Tabriz University of Medical Sciences, Tabriz, Iran; 2 School of Health Management and Information Sciences, Iran University of Medical Sciences, Tehran, Iran; 3 Department of Health Information Technology, School of Allied Medical Sciences, Ahvaz Jundishapur University of Medical Sciences, Ahvaz, Iran

**Keywords:** E-learning, Adoption, Implementation, Faculty member, Tutors, Covid-19

## Abstract

**Background:**

The importance of successful implementation of e-learning, especially since the emergence of the Covid-19 pandemic, has become increasingly apparent to universities. Thus, identifying the effective factors in adopting e-learning in the Covid-19 pandemic is crucial. This study was conducted to identify determining factors in adopting E-learning in healthcare.

**Method:**

This was a descriptive-analytical study in which 143 faculty members from Iran were randomly selected. The faculty members’ intentions, concerning the adoption of e-learning, were assessed by the conceptual path model of integration of unified theory of acceptance and use of technology (UTAUT) and The Task-Technology Fit (TTF).

**Results:**

The results showed that the combination of the two classical theories, UTAUT and TTF, was an appropriate model to explain faculty members’ intention in adopting e-learning. Moreover, the findings showed that technology and task characteristics, task- technology fit, social influences, effort expectancy, performance expectancy and facilitating conditions had direct and significant effect on e-learning adoption.

**Conclusion:**

By presenting a conceptual path model to elucidate users’ behavior in adopting e-learning, this study investigated and identified the key determining factors in adopting e-learning. The findings of the present study can contribute to the design and implementation of e-learning by practitioners, policy makers, and curriculum designers.

## Introduction

As a results of rapid growth of information and communication technologies, electronic learning (e-learning) has amalgamated with the traditional educational structure, causing drastic changes in the landscape of learning by offering various opportunities to explore and develop new ways of delivering educational programs [1,2]. Thus, e-learning has become vastly prevalent and incorporated in teaching and learning programs of many institutions at higher education [3].

Compared to traditional learning environment, e-learning provides interactive and innovative learning reshaping traditional education into a more flexible and efficient one [3,4]. Also, e-learning reduces education time and is highly beneficial in providing cost-effective education which has removed geographical boundaries [4,5].

Considering the importance and the capacity of e-learning, institutions have made major investments in this area, and have strived to integrate and expand e-learning in education programs [4]. E-learning has become an integral part of educational programs in higher education and in most cases, it has completely replaced the traditional teaching approach. E-learning has been welcomed by students and instructors due to its potential and benefits.

However, e-learning has sometimes faced barriers such as user-acceptance and adoption in some institutions where instructors are unwilling to practice e-learning [6]. They believe that e-learning does not cover all aspects of teaching, nor does it support all features of learning [7]. Unfamiliarity with e-learning principles, and the underlying theoretical foundations, technophobia and technical glitches are only some of the challenges unwilling instructors may face when they are asked to use e-learning [8,9]. Also, the available e-content and infrastructure required to support e-learning have not proven to be sufficient for the successful implementation of e-learning systems [4]. Furthermore, it has been reported that the success of e-learning systems depends on learners’ attitudes towards adopting a modern technology [4].”

It is believed that instructors’ attitudes and intention to adopt and use e-learning are critical factors influencing its successful implementation; this has been supported by a large number of empirical studies, conducted in developed countries [9,11,13]. Thus, identifying determining factors in adopting e- learning has been recommended [4]. However, due to the scarcity of the studies addressing the determining factors in adopting e-learning in developing countries, such as Iran, a study should be conducted and effective factors in successful and comprehensive implementation of e- learning should be examined. The findings could aid policy makers, managers, lectures and practitioners in developing strategic policies.

Recently, e- learning has been accompanied by the employment of new and innovative technologies, such as virtual reality (VR) and augmented reality (AR), which have made the process of adoption more complicated. Moreover, many technology adoption models have been developed to help elucidate the factors that can influence the adoption of these technologies [10].

After the spread of the Corona virus in developing countries including Iran, many traditional classrooms at universities were closed, leading to a better understanding of the importance of e-learning. Organizations which had already invested in this area were able to rely on this capability and potential to pursue their educational processes and activities, and the universities that lagged behind, had to struggle the challenges to take an inevitable step forward. It is now clear to everyone that using e-learning is no longer a competitive advantage but a competitive necessity.

Successful adoption of e-learning in medical education depends on identifying and managing the determinant factors. Without having an accurate insight in to the current situation and without identifying the effective factors in the implementation of e-learning, it is unlikely to implement an e-learning program successfully. There are several studies regarding the successful implementation of the e-learning, but we did not find an outstanding study conducted with the combination of UTAUT and TTF models. Thus, we proposed a study which could elucidate and show a comprehensive scenario of e-learning acceptance via analyzing the variables of task- technology fit and UTAUT variables. This descriptive –analytical study was conducted to identify determining factors in adopting e-learning in medical education in Iran.

## Research model and hypothesis

Among technology acceptance and adoption models which explain end-users’ behavior in adopting and accepting a new technology or product, UTAUT has attracted particular attention. UTAUT is an extension of Technology Acceptance Model (TAM), which was initially proposed by Venkatesh et al. [11]. This model enables us to survey dependent variables including behavioral intention by estimating the effect of four determining variables i.e. performance expectancy (PE), effort expectancy (EE), social influence (SI), and facilitating conditions (FC) [10,11].

The Task-Technology Fit (TTF) model is another practical model which has been widely applied as a theoretical model to explain how a new technology could lead to performance, evaluation of adoption impact, and assessment of the relation between the task and technology characteristics. This model was developed by Goodhue and Thompson in 1995, and explains how both the task characteristics and technology characteristics affect the task-technology fit, which ultimately determine end-user performance and utilization [12,13]. In fact, TTF discusses that a user adopts an information technology when it fits his/ her tasks and improves his/her performance [13].

“TTF has been widely used and combined with other models such as TAM to explain user adoption of an information technology” [13].When UTAUT and TTF are put together, they can create an integrated framework for elucidating and predicting end-users’ behavior and intention in acceptance and adoption of new technologies.

The current study was conducted on the basis of the combined UTAUT and TTF in order to recognize effective factors in adopting e-learning by faculty members in medical education. Hence, by providing a clear and logical explanation of the current situation, effective factors for using e-learning by instructors are identified.

Zhou et al. [13] found that both technology and task characteristics determine user adoption by effecting the task- technology fit [13]. Also, Zhou et al. [13] and Wu and Chen [12] remarked that task- technology fit has obvious and significant effect on end-user adoption. Based on the results of previous studies, the hypothesis H1 to H3 were put forth as:

- H1: Technology characteristics affects task-technology fit.
- H2: Task characteristics affects task- technology fit.
- H3: Task technology fit affect Intention to adopt e-learning.

Chang et al. [14] investigated the factors influencing the adoption of medical apps by patients. They employed a theoretical model that combines the unified theory of acceptance and use of technology with the concept of technology readiness. They have reported that PE, EE, and SI have significant positive effects on the behavioral intention of individuals, but technology readiness has significant negative effects, indicating that technology readiness moderates the association between PE and behavioral intention [14]. Chao [15] has also reported that behavioral intention is significantly and positively influenced by satisfaction, trust, PE, and EE in adopting mobile learning by students.

Maldonado, Alrawashdeh et al., Tan, Usoro et al., and Abdekhoda et al. [16–20] have reported that SI has a direct and significant effect on new technology adoption. Alrawashdeh et al., Tan, Nassuora, Abdekhoda et al., Abdekhoda et al., and Zhou et al. [6,16,18,19,21] have found that EE has a direct and significant effect on end-users’ intention in adopting e-learning. Abdekhoda et al. and, Zhou et al., Alrawashdeh et al., Tan et al, and Echeng et al. have discussed that PE has a direct and significant effect on users’ behavior intention in adopting a new technology [6,13,16,18,19,22]. Based on current research and to identify the effective factors in adopting e-learning in Covid-19 era, the fallowing hypothesis were put forth:

- H4: Social influences affect Intention to adopt E-learning.
- H5: Effort expectancy affect Intention to adopt E-learning.
- H6: Performance expectancy affect Intention to adopt E-learning.
- H7: Facilitating condition affect Intention to adopt E-learning.

**Fig. 1:**
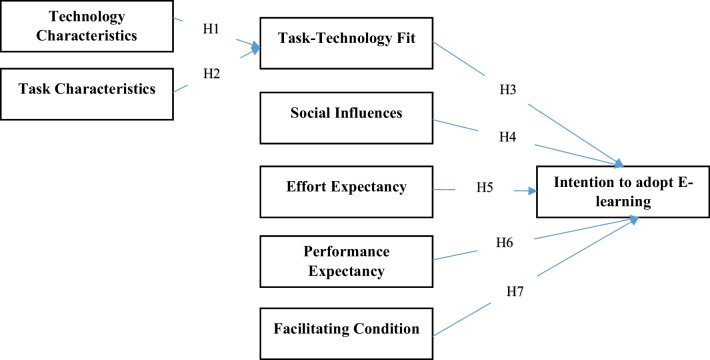
The proposed research model

The following hypothesis which are summarized in Fig. 1 were put forth in this study.

## Martials and methods

### Study design and participants

This research was a descriptive-analytical study. The research population consisted of faculty members of Ahwaz University of Medical Sciences. This university was selected as the setting of the research because on the basis of Iran ministry of health report, it has obtained the first rank in e-learning implementation in medical education after the Covid-19 pandemic. On the basis of Morgan’s table 143 of the faculty members were selected as the sample.

### Sample

The research population included faculty members of Ahwaz University of Medical Sciences, 143 of whom were selected as the sample based on Morgan’s table. The non-probability sampling technique with a combination of convenient sampling methods was applied to obtain responses from the study population. The faculty members were included if were affiliated to AUMS, had the willingness to cooperate in the study via a self- declaration form, and were familiar with e-learning.

### Instrument

A researcher-made questionnaire was developed as a data collection tool based on the literature related to e-learning adoption and implementation such as Wu and Chen [12] article. In order to check the validity of the questionnaire, a validation form was provided, in which three aspects of relevance, clarity and simplicity were considered for each item. The validation forms were distributed among 10 specialists in the field of medical education. The content validity index (CVI) and content reliability ratio (CVR) were considered for each item and the items receiving a low score were eliminated. In order to determine the reliability, the questionnaire was distributed among 30 instructors, and then the internal correlation among the items was measured and Cronbach’s alpha was obtained (0.92). Finally, the questionnaire was developed with nine variables and 25 items, including the variables of demographic information, Social Influence (SI), Effort Expectancy (EE), Performance Expectancy (PE), Behavioral Intention (BI), Facilitating Conditions (FC), Technology Characteristics (TEC), Task Characteristics (TAC), and Task Technology Fit (TTF). Survey questions were used to measure the variables as presented in Table 1. Data were collected by distributing questionnaires among instructors at their offices or sending emails.

**Table 1:** Measurement items examined in the model

Constructs	Items number	Items
Social influence (SI)	1	People who are important to me think that I should use LMS
	2	People who influence my behavior think that I should use LMS
Effort expectancy (EE)	3	I can quickly and easily utilize LMS
	4	I can skillfully use a LMS
	5	I find LMS easy to use
	6	I think learning to operate LMS is easy for me
Performance expectancy (PE)	7	I feel LMS are useful in teaching and learning
	8	Using LMS enables me to increase teaching productivity
	9	Using LMS enhances my engagement in education
	10	If I use LMS, I will increase my authority of managing education
Behavioral intention (BI)	11	I expect using LMS could have importance progress in learning
	12	I can develop a habit to use LMS soon
	13	In the future, I will often use LMS
Facilitating conditions (FC)	14	I have the necessary resources to use LMS
	15	I have the necessary knowledge and skill to use LMS
	16	If I have difficulty using LMS, there will be experts to help me
Technology characteristic (TEC)	17	LMS provide ubiquitous service in education
	18	LMS provide real-time service
	19	LMS provide reliable service
Task characteristic (TAC)	20	I need to manage my education easily, anytime anywhere
	21	I need to access my learning contents anytime anywhere
	22	I need to evaluate my student’s progress, anytime anywhere
Task technology fit (TTF)	23	In my education, the functions of LMS are enough
	24	In my health management, the functions of LMS are appropriate
	25	Generally, the functions of LMS completely meet my demand

### Data analysis

Statistical analysis was performed by using the SPSS_16_ software package. Cleaning of data was carried out to ensure that there were no missing or abnormal data by running frequencies and descriptive statistics. Data were presented by using descriptive statistics including frequencies and percentages for categorical variables, means and standard deviations for continuous variables. Spearman correlation analysis and regression tests were used for assessment of the relationship among quantitative variables.

## Results

The demographic analysis showed that from 143 participants, 51 percent (73 person) were females and 49 percent (70 person) were males. The data also showed that more than 38 percent were between 41and 48 years old, while 35 percent of them were between 49 and 56. Also we found that 47 percent of participants had between 16 and 23 years of teaching experiences. Regarding experiences in using e-learning, the data showed that 60 percent had between 1and 3 years of experience in adopting e-learning.

Table 2 shows the correlation matrix of the proposed model’s variables. Data presented in the table shows that there is a positive and significant correlation between technology and task characteristics on one hand and task- technology fit on the other hand. The data also show that task- technology fit has a positive and significant correlation with UTAUT. Overall, there was a positive and significant correlation between the two model variables.

**Table 2:** Correlation matrix of the proposed model’s variables

Variables	Technology characteristics	Task characteristics	Task-technology fit	Social influences	Effort expectancy	Performance expectancy	Facilitating condition	Intention to adopt E-learning
Technology characteristics	1	0.678**	0.652**	0.118	0.246**	0.223**	0.542**	0.381**
Task characteristics	0.678**	1	0.525**	− 0.023	0.251**	0.377**	0.644**	0.452**
Task-technology fit	0.652**	0.525**	1	0.113	0.279**	0.160	0.386**	0.244**
Social influences	0.118	− 0.023	0.113	1	0.273**	0.192*	0.75	0.262**
Effort expectancy	0.246**	0.251**	0.279**	0.273**	1	0.496**	0.451**	0.464**
Performance expectancy	0.223**	0.377**	0.160	0.192*	0.496**	1	0.375**	0.679**
Facilitating condition	0.542**	0.644**	0.386**	0.075	0.451**	0.375**	1	0.423**
Intention to adopt E-learning	0.381**	0.452**	0.244**	0.262**	0.464**	0.679**	0.423**	1

**Correlation is significant at the 0.01 level (2-tailed)

*Correlation is significant at the 0.05 level (2-tailed)

**Fig. 2:**
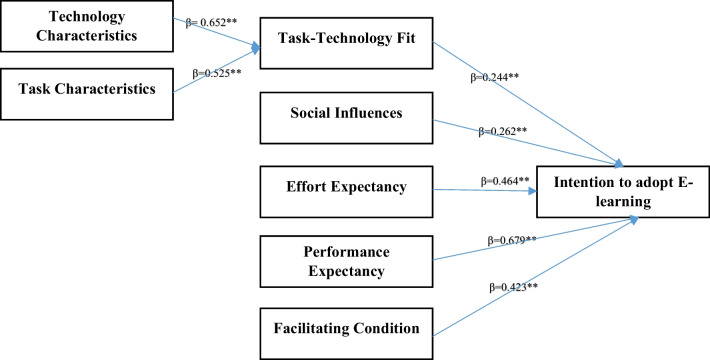
The validation of the proposed research model. **Correlation is significant at the 0.01 level (2-tailed))

Figure 2 shows the validation of the proposed research model. As this figure shows technology and task characteristics have positive and significant effect on task- technology fit. Also, this figure presents independent variables including task-technology fit, SIs, EE, PE, and FC which have a direct and significant effect on instructors’ intentions to adopt e-learning.

A summary of the hypotheses testing results of the standardized path coefficients and path significances is presented in Table 3. The table shows that all of the paths are significant in the expected direction.

**Table 3:** Model path analysis

The hypothesis	Path coefficient	*P* value	Support
H1: technology characteristics affect task-technology fit	0.652	0.000	Yes
H2: task characteristics affect task- technology fit	0.525	0.000	Yes
H3: task technology fit affect Intention to adopt E-learning	0.244	0.000	Yes
H4: social influences affect Intention to adopt E-learning	0.262	0.000	Yes
H5: Effort expectancy affect Intention to adopt E-learning	0.464	0.000	Yes
H6: performance expectancy affect Intention to adopt E-learning	0.679	0.000	Yes
H7: facilitating condition affect intention to adopt E-learning	0.423	0.000	Yes

## Discussion

This study was conducted to discover instructors’ behavioral intention in adopting e-learning during the Covid-19 pandemic, on the basis of the combination of two classical theories of UTAUT and TTF. This study investigated the effect of technology and task characteristics on task- technology fit and also examined the effect of task- technology fit, SI, EE, PE and FC as external factors on faculty members’ intention in adopting e-learning.

Concerning the association between technology characteristics with task-technology fit, the standard coefficient of technology characteristics and task- technology fit was found to be 0.652 (*p* = 0.001). Thus, H1 was supported, indicating that technology characteristics affect task-technology fit in adopting e- learning. The results also showed that the standard coefficient of task characteristics and task- technology fit was found to be 0.525 (*P* = 0.001); thus, H2 was supported, showing task characteristics affect task-technology fit in adopting e-learning. In line with the results of the current study, Zhou et al. [13] found that both technology and task characteristics have significant effect on task-technology fit, thereby determining user adoption. This finding supports previous research results. Pal and Patra ^[[[[23]]]]^ conducted a study in which a combination of two classical theories, TAM and TTF, was used to elucidate university students’ perception of video-based learning in times of COVID-19. They found that technology characteristics has direct and significant effect on task technology fit. Also, they acknowledged that task technology fit has direct and significant effect on actual use of video-based learning by effecting on both perceived ease of use and perceived usefulness [23]. Thus, technology and task characteristics should be considered in adopting a new technology, in this case e-learning. This is based on the assumption that users will adopt a new technology according to the fit between the technology characteristics and task requirements [13].

In terms of the relationship between task- technology fit with intention to adopt e-learning, the standard coefficient of task- technology fit and intention to adopt e-learning was found to be 0.244 (*P* = 0.001); thus, H3 was supported, indicating task- technology fit affects instructors’ intentions to adopt e-learning. Wu and Chen [12] found that task-technology fit has a direct and significant effect on perceived usefulness, which further affects user attitude and the intention to adopt Massive Open Online Courses (MOOCs). Similar with the results of this study, Zhou et al. [13] acknowledged that task-technology fit had significant effect on end-user adoption. Task-technology fit is defined as a matter of how the competency of a new technology matches with the tasks that the person must perform, which is a determining factor in explaining job performance levels [24]. Furthermore, based on the finding of this study and other similar studies, task- technology fit is a major factor in adopting new technologies such as e-learning. Users welcome new technologies that can help them perform their tasks easily; however, the technology should be simple and comprehensible and effective. Thus, this issue should be addressed when successful and comprehensive implementation of e-learning is in progress.

As for the relationship between SI and intention to adopt e-learning, the results show that SI has a direct and significant effect on intention to adopt e-learning (β = 0/262, *p* = 0.001); thus, H4 was supported. This findings is in line with the studies of Maldonado, Alrawashdeh et al., Tan, Usoro et al., and Abdekhoda et al. [16–20], who have reported that SI has a direct and significant effect on new technology adoption. The ways people of a society exert effort to form values, beliefs, perception, intention, attitude and behavior are Sis [25]. Thus, values, beliefs, perception, intention, attitude and behavior in the society affect the adoption of e-learning.

Regarding the association between EE and the intention to adopt e-learning, the standard coefficient of EE and the intention to adopt e-learning was found to be 0.646 (*P *= − 0.001); thus, H5 was supported; suggesting that EE affects instructors’ intention to adopt e-learning. Likewise, Alrawashdeh et al., Tan, Nassuora, Abdekhoda et al., Abdekhoda et al., and Zhou et al. [6,16,18,19,21] found that EE had a direct and significant effect on end-users’ intention in adopting e-learning. “Effort expectancy is defined as user perception of how they can use a technology easily” [26]. Thus, ease of use of e-learning should be considered by designers and mangers, because the findings of the current study and pervious research suggest that users adopt systems which have considerable efficacy and can be used easily.

Concerning the relationship between PE and intention to adopt e-learning, the results show that PE has a direct and significant effect on the intention to adopt e-learning (β = 0/679, *p* = 0.001); thus, H6 was supported. This finding supports the findings of previous studies, which have shown that PE has a direct and significant effect on users’ intention behavior in adopting a new technology [6,13,16,18,19,22]. Performance expectancy is defined as “the expected impact of a technology’s functional advantage even in uncertain conditions” [27]. Users’ acceptance of any new system such as e-learning takes place, when users perceive that the new system has considerable advantages and promotes their performance.

Finally, regarding the association between FCs and intention to adopt e-learning, the standard coefficient of FCs and intention to adopt e-learning was found to be 0.423 (*P* = 0.001; thus, H7 was supported, indicating that FCs affect instructors’ intention to adopt e-learning. Similarity, Alrawashdeh et al. [18], Echeng et al. [22], Nassuora [21], Abdekhoda et al. [16], and Zhou et al. (2017) have reported that FCs have a significant effect on end users’ perception when applying a new technology is considered.

This was a descriptive analytical study to investigate the determining factors in adopting e-learning in Covid-19 pandemic. This study presented a comprehensive scenario of the determinant factors in successful adoption of e-learning, based on an integration model of UTAUT and TTF. The combination of these two models to elucidate the faculty members’ intention toward e-learning adoption in Covid-19 pandemic, is the distinction of this research from other similar studies, indicating the combination of UTAUT and TTF can be a comprehensive and powerful model to identify the determinant factors of e-learning. This idea can be used in futures studies regarding new technology adoption.

Also, this study clearly identified effective and prominent factors in adopting e-learning which should be considered as the second distinctive feature of this study. We found that technology and task characteristics have a direct and significant effect on task- technology fit, which further determines instructors’ intention to adopt e-learning. This finding can help service providers to improve the task-technology fit of e-learning. Meanwhile, we found that SIs, EE, PE and FCs have considerable effects on instructors’ intention to adopt e-learning. These factors should be taken into consideration by providers and policy makers to promote e-learning in developing countries.

However, this study has the fallowing limitations that should be addressed in future studies. First, the setting of this study is limited to faculty members of Ahwaz University of Medical Sciences, and due to some barriers we were not able to include all faculty members of Iran’s ministry of health. If we had included faculty members of all Iranian universities of medical sciences as the study population, we would have been able to provide a more comprehensive picture of the current situation. Second, the intention to adopt e-learning is dynamic and may change constantly, but this study was carried out as a cross-sectional study. Finally, the items of the questionnaire were selected on the basis of instructors’ selection which might have a self-selection bias.

## Conclusion

This study investigated the key determining factors of e-learning adoption according to the integrated model of UTAUT and TTF in a top rank university of Iran, Ahwaz University of Medical Sciences. We examined successful and comprehensive implementation of e-learning in Covid-19 pandemic and identified effective factors in the adoption of e-learning. The significant finding of the study is that identifying determining factors in the adoption of e-learning is necessary if we attempt to have a successful implementation of an e-learning system. The findings of the study have valuable contribution to the design and implementation of e-learning systems by policy makers, administrative bodies, and curriculum designers. In-depth analysis of the determining factors, with the inclusion of a large population are recommended for the future studies.

## Data Availability

The datasets used and/or analyzed during the current study are available from the corresponding author on reasonable request.
